# Educational Intervention for the Management of Nonspecific Lower Back Pain in Nonprofessional Caregivers (TRANSFE Program): A Quasi-Experimental Study

**DOI:** 10.3390/nursrep14030118

**Published:** 2024-06-27

**Authors:** Víctor Ortiz-Mallasén, Eloy Claramonte-Gual, Águeda Cervera-Gasch, Desirée Mena-Tudela, María Jesús Valero-Chillerón, Laura Andreu-Pejó, Irene Llagostera-Reverter, Víctor Manuel González-Chordá

**Affiliations:** 1 Nursing Department, Jaume I University, Avda Sos Baynat s/n, 12071 Castellón de la Plana, Spain; cerveraa@uji.es (Á.C.-G.); dmena@uji.es (D.M.-T.); chillero@uji.es (M.J.V.-C.); pejo@uji.es (L.A.-P.); llagoste@uji.es (I.L.-R.); vchorda@uji.es (V.M.G.-C.); 2 Department of Health in Castellón, Valencian Health System, Avda Benicassim, 128, 12004 Castellón de la Plana, Spain; claramonte_elo@gva.es

**Keywords:** low back pain, caregivers, nursing, evaluation of the efficacy-effectiveness of interventions, caregiver burden

## Abstract

Nonspecific lower back pain is one of the main health issues experienced by nonprofessional caregivers of dependent individuals. The repetitive movements and efforts made by caregivers to assist dependent individuals are associated with the onset of this lower back pain. The main objective of this study was to assess the effectiveness of an educational intervention for the management of nonspecific lower back pain in nonprofessional caregivers of dependent individuals (TRANSFE program). The secondary objectives were to (i) evaluate the effectiveness of the TRANSFE program on other variables (caregiver burden, perceived social support, and health-related quality of life), (ii) obtain the sociodemographic profile of the sample, and (iii) determine the baseline of the study variables. A quasi-experimental study with post-intervention measurements at 3 months was conducted. Thirty-six nonprofessional caregivers of dependent individuals participated in this study. The presence of lower back pain (back pain index), low back pain (visual analogue scale), disability due to low back pain (Oswestry disability index), perceived social support (Duke-UNK scale), caregiver burden (Zarit burden scale), and health-related quality of life (EuroQol-5D) were assessed. The intervention significantly improved all the studied variables related to lower back pain (*p* < 0.001). The intervention was effective on other variables related to nonprofessional caregiving such as caregiver burden, perceived social support, and health-related quality of life, albeit with moderate results. An educational intervention for lower back pain experienced by caregivers of dependent individuals was effective in reducing lower back pain and addressing caregiver burden, perceived social support, and health-related quality of life. This study was registered retrospectively on the Open Science Framework platform on 20 June 2024, with the registration number 10.17605/OSF.IO/K7WTE.

## 1. Introduction

Lower back pain, which is more than a personal experience, has emerged as a significant challenge in the context of musculoskeletal disorders. Its presence not only affects individuals at a personal level but also projects itself as a widespread public health issue [1]. This condition, recognised globally, does not discriminate based on age, nationality, or profile, exerting its impact in a generalised manner, leading to considerable economic and social consequences [2]. The magnitude of the problem becomes evident as lower back pain currently stands as the leading global cause of years lived with a disability. This underscores the urgency and critical importance of developing effective strategies for both the management and prevention of lower back pain [3].

The complexity and breadth of this phenomenon highlight the need to address it from multiple perspectives, ranging from individual clinical care to public health policies. This comprehensive approach is necessary to mitigate the impact of lower back pain and enhance the quality of life of those experiencing it. The intersection of the individual and the collective in this context emphasises the importance of a diverse approach involving healthcare professionals, researchers, policymakers, and society as a whole [3].

The clinical definition of lower back pain, which is identified as a syndrome characterised by discomfort in the lower segment of the spine [4], establishes a fundamental basis for understanding this condition from a medical and anatomical perspective. However, the magnitude of its impact significantly increased when its standardised point prevalence was examined.

Globally, the point prevalence of lower back pain is estimated to be 7.50% [5], indicating the frequency with which this condition affects the population in various contexts and regions. However, it is crucial to note that reality varies according to specific sociodemographic conditions. For the Spanish population, the figure increases to 10% [5,6], demonstrating an even more significant burden in this particular context.

According to the National Health System report from 2017 (published in 2019), lower back pain was the second most common chronic health problem in the Spanish population, affecting 18.5% of the population. It occurs more frequently in women than in men, with a ratio of 1.5 (14.7% in men and 22.1% in women). The highest incidence of lower back pain is observed in the decade between 45 and 55 years of age, being the main cause of work disability in people under 45 years old [7].

The increased prevalence of lower back pain in the Spanish population suggests the presence of specific factors that may contribute to its development in this country. These factors may include cultural and socioeconomic elements or even lifestyle variations that can influence the incidence and experience of lower back pain. In regions where intense physical labour without proper lifting techniques prevails, an increased risk of back problems is observed. Additionally, the availability of health and education resources is crucial, as individuals with low incomes may have less access to quality healthcare and preventive measures for lower back pain. Working conditions in low-income jobs also contribute to the issue, with fewer ergonomic protections and more strenuous physical labour. Conversely, sedentary lifestyles and obesity add strain to the spine, while regular physical activity can strengthen back muscles and improve overall spinal health [6]. Considering these local differences is essential for developing prevention and treatment strategies, underscoring the importance of addressing lower back pain in a contextualised and tailored manner according to the particular characteristics of the target population [2].

The most common type of lower back pain is nonspecific, highlighting the intrinsic complexity of its diagnosis as its pathoanatomic cause cannot be precisely determined [8]. This clinical nuance adds a challenge to the understanding and treatment of this condition because its origin cannot be easily attributed to a specific identifiable cause.

However, various risk factors are associated with the development of lower back pain, which broadens our understanding of its possible determinants. These factors include female sex, sedentary behaviour, obesity, and smoking [4,9,10,11,12], each contributing uniquely to the complexity of the phenomenon. Nevertheless, occupational elements have emerged as crucial determinants of the development of lower back pain [12].

Specific or repetitive overexertion related to the mobilisation and transfer activities of dependent individuals establishes a direct connection between lower back pain and caregiving tasks. This finding suggests that physical work associated with caregiving, including repetitive movements and load transfers, may play an essential role in the genesis of lower back pain [13,14]. This information is crucial for guiding preventive and therapeutic strategies, emphasising the importance of addressing not only general risk factors but also specific conditions associated with occupational responsibilities, especially in the realm of caring for dependent individuals [13].

Nonprofessional caregivers, defined as individuals who undertake the care of others without specific training or financial compensation for their efforts, play a crucial role in the care system for dependent individuals [15]. The proportion of this type of caregivers in the different European states ranges from 20% to 44% of the entire population, with intensive caregivers (defined as those who dedicate more than 11 h per week to informal care) ranging from 4% to 11% [16].

Women aged 45–60 are the main providers of non-professional care in all European countries, including Spain. When considering intensive caregivers, women also constitute the majority, with a higher percentage in southern countries. This suggests that in those countries where the State does not support caregiving and leaves the responsibility to families, there are fewer people willing to do so, and those who end up doing it do so with greater intensity. This is key to discussing the “caregiver burden” and gender inequalities. This group, often composed of close relatives, becomes the connective tissue that sustains and provides continuous care to those who depend on them [17].

In Spain, 90% of nonprofessional caregivers are direct family members, highlighting the importance of family networks in supporting and caring for dependent individuals [18]. This commitment translates into significant daily time dedication to the care of loved ones and providing assistance in basic activities of daily living [19]. These activities range from assistance with mobility to participation in essential daily tasks and form an integral part of the well-being and quality of life of dependent individuals [20].

However, this selfless and seemingly inexhaustible dedication has consequences. The intrinsic burden associated with non-professional caregiving manifests in the health of these caregivers, becoming a concern for healthcare professionals. Previous studies [11,15,17], have suggested that caregivers with lower levels of education may face greater risks due to a potential lack of knowledge about safe caregiving techniques and injury prevention. Additionally, age can also play a significant role, as older caregivers may experience a decline in physical strength and increased frailty, heightening their vulnerability to caregiving-related injuries. Other factors, such as workload, lack of social support, and unsafe working conditions, can also contribute to the risk of injuries among caregivers.

Lower back pain is one of the most prominent health issues in this population, reflecting the physical and emotional burden to which caregivers are subjected [14,21,22,23]. This phenomenon highlights the urgent need to understand and address the implications of nonprofessional caregiving, recognising the vital importance of implementing support strategies and resources to preserve the health and well-being of those providing this type of care.

In Spain, multiple interventions have been implemented for this population [24], yielding encouraging results [25]. These interventions mostly focus on mitigating the negative consequences associated with the increased emotional burden experienced by nonprofessional caregivers [24]. Such interventions are generally effective in addressing emotional and psychological aspects linked to family caregiving of dependent individuals [25].

However, it is important to note that the existing literature reflects a notable gap in addressing physical problems such as lower back pain, which significantly affects this group of caregivers. The limited availability of research focusing on lower back pain suggests a gap in scientific knowledge and intervention strategies to address the physical aspects of nonprofessional caregivers [26].

It is encouraging to note that the available research suggests a positive effect of these interventions focused on lower back pain. A reduction in pain intensity and an improvement in functional limitations were observed, which translated into a positive impact on the quality of life of the caregivers. These findings indicate the need to expand and deepen interventions that specifically address physical problems such as lower back pain to improve the overall health of nonprofessional caregivers [25,26].

Therefore, the importance of developing more holistic intervention strategies, addressing both the emotional and physical aspects of caregiving, is emphasised. By doing so, more comprehensive and effective tools can be provided to support nonprofessional caregivers, enabling them to cope with the inherent challenges of their work in a more integrated and sustainable manner [14,26,27].

In light of this context, the main objective of the present study was to evaluate the effectiveness of an educational intervention for the management of nonspecific lower back pain in nonprofessional caregivers of dependent individuals (TRANSFE program). The secondary objectives were to (i) assess the effectiveness of the TRANSFE program in relation to other variables (burden, perceived social support, and health-related quality of life), (ii) obtain the sociodemographic profile of the sample, and (iii) understand the baseline of the study variables.

## 2. Materials and Methods

### 2.1. Design and Setting

A quasi-experimental prospective study was conducted with a single study group and repeated measurements at 3 months post-intervention between January 2017 and October 2018. This study was conducted in the Health Department of Castellón in the Valencian Community, Spain. This health department includes both primary and hospital care, with two general hospitals (837 beds) and 25 health centres providing public healthcare assistance to approximately 300,000 people in the province of Castellón, Spain. This study was registered retrospectively on the Open Science Framework platform; it can be accessed through the link https://osf.io/k7wte/ (accessed on 20 June 2024). The registration number is 10.17605/OSF.IO/K7WTE.

### 2.2. Population

The study population consisted of nonprofessional caregivers of dependent individuals who met the following inclusion criteria: (i) being a nonprofessional caregiver of dependent individuals aged 18 years or older, (ii) having been a caregiver for at least 6 months, and (iii) experiencing nonspecific lower back pain. The exclusion criteria included (i) not being the primary caregiver, (ii) having lower back pain of known pathoanatomic origin, and (iii) not providing informed consent.

### 2.3. Sample Size Calculation

The sample size was estimated to evaluate the effectiveness of the intervention based on lower back pain (measured using the Oswestry disability index), assuming an infinite population for the calculation. A two-sided test with an alpha risk of 0.05 and a beta risk of 0.2 was considered, requiring at least 32 participants to detect a difference equal to or greater than 16.1 points on the Oswestry disability index, which ranges from 0 to 50 points. A common standard deviation of 15.9 points and a correlation coefficient between the initial and final measurements of 0.5 were assumed, and a follow-up loss rate of 10% was estimated. Therefore, a final sample size of 36 participants was required.

### 2.4. Educational Intervention: TRANSFE Program

The intervention, designed by the principal investigator and based on the best available evidence, aimed to provide specific training on the mobilisation and postural transfers of dependent individuals. The educational intervention sought to provide knowledge that would enable caregivers to perform these manoeuvres ergonomically, with the goal of improving lower back pain associated with their repeated performance.

The content was selected based on similar programs [26,27,28,29]. It consisted of a 120 min session with four parts: (i) postural hygiene in activities of daily living, (ii) basic self-stretching exercises, (iii) techniques for mobilising dependent individuals, and (iv) teaching of ergonomic transfer manoeuvres. The session’s development was systematic. Theoretical information was first provided through an oral presentation with computer support and a PowerPoint^®^ presentation. Subsequently, practical exercises were conducted to acquire skills in mobilisation and transfer manoeuvres.

The intervention was individualised and conducted in the caregivers’ homes, allowing information to be personalised to the caregiver’s characteristics (physique, age, muscle strength, previous caregiving skills, etc.), dependent person’s characteristics (physique, level of dependence, etc.), and caregiving environment. The adaptation of the content was carried out by the researcher who delivered the intervention, followed a checklist, and was based on their clinical experience. To reduce the possibility of bias, all the interventions were conducted by the same researcher.

### 2.5. Variables and Measurement Instruments

Sociodemographic variables were considered for both the caregiver and dependent person, including sex, age, educational level, relationship, and employment status. The main outcome variables were lower back pain (back pain index) [30], level of back pain (visual analogue Scale) [31], and disability due to back pain (Oswestry disability index) [32]. These variables are appropriate for assessing the specific impact of the program, as indicated in other studies [27,33].

To study the possible impact of the intervention on other aspects, the following secondary outcome variables were selected: caregiver burden (Zarit burden scale) [34], social impoverishment (Duke-UNK scale) [35], and health-related quality of life (EuroQol-5D) [36]. Table 1 presents the characteristics and psychometric properties of the instruments used in this study.

### 2.6. Data Collection

The recruitment of caregivers participating in the study was conducted by primary care nurses from health centres affiliated with this study between January 2017 and January 2018. Nurses offered participation to the caregivers of dependent individuals attending clinics who met the selection criteria. All participants were provided with an information sheet outlining the study’s characteristics and purpose along with an informed consent form.

Data were collected using an information collection notebook that included a section on sociodemographic data (obtained through personal interviews and electronic medical records) and a section on the measurement instruments of the scales used (self-administered by the participants). Data were collected at the participants’ homes in two phases: prior to the intervention (baseline or pre-intervention measurement) and at three months (post-intervention measurement). This process was performed by the same investigator in all cases.

### 2.7. Data Analysis

The significance level was set at 5%, considering differences with *p* < 0.05. All analyses were performed using the statistical software IBM-SPSS v.22. First, a descriptive analysis of the variables was conducted based on their characteristics. Means and measures of dispersion were calculated for continuous variables and frequency distributions and proportions were used for categorical variables.

The effectiveness of the intervention was assessed based on the decrease in the scores on the measurement instruments for the presence and level of lumbar pain (calculated using a Wilcoxon test for related measures). Improvement in the agreement analysis for the variable of incapacity due to lumbar pain was also assessed (where the Kappa coefficient was used as a measure of association, as indicated by some authors such as Mast [37]).

The Kolmogorov–Smirnov test was used to determine if the data followed a normal distribution. Quantitative variables with a normal distribution were compared using the Student’s *t*-test, and in cases of non-normality, the Mann–Whitney U test was used. To compare differences between pre- and post-intervention moments, Student’s t-test was used, and in cases of non-normality, the Wilcoxon test was used. For qualitative analysis, contingency tables were generated and statistical significance was analysed using the chi-square test.

### 2.8. Ethical Considerations

This study was approved by the Clinical Research Ethics Committee of the Castellón Health Department (registration number: 10/2016). All participants signed an informed consent form and were informed of their rights regarding data confidentiality (Organic Law 15/1999 of 5 December on the Protection of Personal Data) and were given the opportunity to withdraw from the study at any time. The recommendations outlined in the International Ethical Guidelines for Biomedical Research Involving Human Subjects, approved in 2002 by the Council for International Organisations of Medical Sciences (CIOMS), and the Declaration of Helsinki of the World Medical Association in 2008, were followed.

## 3. Results

### 3.1. Sociodemographic Profile of the Sample

**Figure 1:**
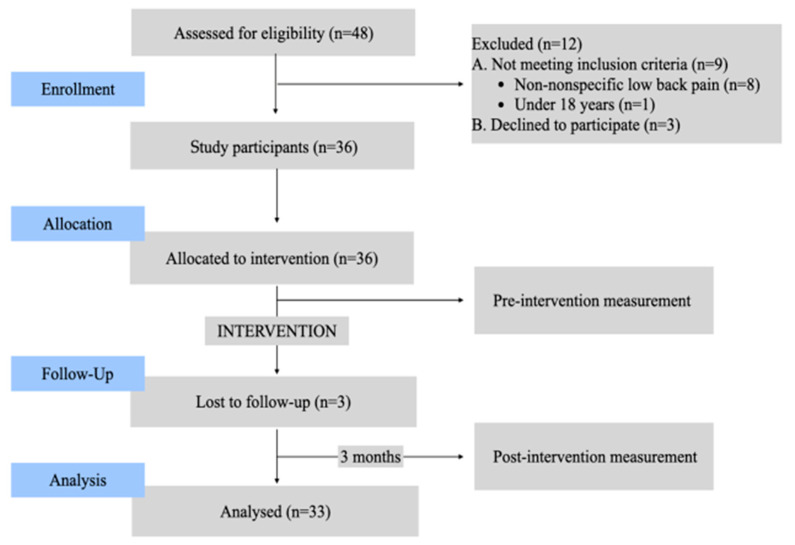
Flowchart of study participants.

Initially, 48 nonprofessional caregivers were recruited, of which 12 were excluded from the study before starting the intervention for not meeting the selection criteria (18.7%; n = 9) or because they declined to participate at the last moment (6.25%; n = 3). Finally, 36 caregivers responsible for 39 dependent individuals were included in the study. There were three losses during the study period (Figure 1).

Table 2 shows the sociodemographic and occupational characteristics of the study participants. It can be observed that, in the case of caregivers, the majority were female (72.2%; n = 26), with a mean age of 60.03 ± 10.30 years. Similarly, in the group of dependent individuals, women also predominated (61.5%; n = 24), with a majority being over 65 years old (79.75 ± 9.92 years). According to Barthel, these dependent individuals exhibit a moderate to severe level of dependency in more than 60% of the cases. However, a significant proportion of nonprofessional caregivers (over 80%) had been performing their roles for more than two years.

### 3.2. Baseline Status of Nonprofessional Caregivers

Table 3 shows the results of the pre-intervention measurements for the baseline data of most variables collected in this study. The participants in the sample had lumbar pain with an average score of 7.12 ± 3.67 points on the measurement instrument, corresponding to a mean pain level of 5.69 ± 2.65 points on a visual analogue scale from 0 to 10. Of the caregivers, 41.7% (n = 15) reported a moderate level of disability due to lumbar pain on the Oswestry scale, 30.6% (n = 11) reported severe disability, and the remaining 27.8% (n = 10) reported minimal disability.

In the caregiver burden variable, 61.1% (n = 22) of participants reported experiencing intense burden, while 38.9% (n = 14) experienced mild burden, yielding a baseline mean of 71.91 ± 21.28 in the test used. Regarding the social support perceived by the participants, half (50.0%; n = 18) were seen as having received little support from their closest environment before the intervention.

Regarding health-related quality of life, caregivers indicated that the dimensions in which they experienced the most problems were anxiety (77.8%; n = 28), pain (63.9%; n = 23), mobility (47.2%; n = 17), activity (30.6%; n = 11), and care (11.1%; n = 4). When assessing how they perceived their overall health compared to a year earlier, 52.8% (n = 19) believed it was worse, while the rest were divided into those who saw it as the same (39.9%; n = 14) and those who saw it as better (8.3%; n = 3). Finally, participants reached a baseline mean of 5.94 ± 6.00 points on a 0–10 on the visual analogue scale, which is included in this instrument to assess current health status.

### 3.3. Effectiveness of the TRANSFE Program for Managing Lower Back Pain

After the intervention, the previously obtained mean value in the variable of lower back pain presence was significantly reduced to 4.24 ± 3.12 points, showing a statistically significant decrease (*p*-value < 0.001). Regarding the level of lower back pain, a similarly notable decrease in mean values was observed after the intervention, decreasing to 3.87 ± 2.49 points, with a *p*-value < 0.001 (Table 4).

Three of the caregivers initially involved did not answer the Oswestry disability index for measuring disability due to lumbar pain after the intervention. When analysing the matches in the cross-tabulation, it is observed that out of the 11 cases with an “intense” rating at baseline, 9 showed improvement after the intervention, with 2 of them receiving a “minimal” rating. Of the 15 caregivers with an initial “moderate” rating, 6 decreased to “minimal” (K = 0.275) (Table 5).

### 3.4. Effectiveness of the TRANSFE Program Compared with Other Variables

The baseline mean for the caregiver burden variable decreased to 55.54 ± 10.11 at 3 months post-intervention, showing statistical significance of the intervention in this aspect (*p*-value 0.002), as observed in Table 6. Similarly, the perceived social support variable showed statistically significant improvements in the sample (*p*-value < 0.001), with the mean increasing from 32.24 ± 7.80 at the pre-intervention measurement to 35.72 ± 6.29 at the measurement taken at 3 months.

When analysing the results in the five dimensions of the EuroQol-5D scale for the variable health-related quality of life, a concordance analysis was conducted, finding the lowest agreement at 3 months post-intervention in the dimensions of “pain” (K = 0.153), “anxiety” (K = 0.084), and “activities” (K = 0.212). Regarding how caregivers perceived their health compared to the last 12 months, a notable finding was K = 0.429, corresponding to a significant improvement in responses given three months post-intervention compared with baseline. However, no significant *p*-value was found (*p* = 0.068) in caregivers’ self-rated health scores at three months post-intervention (Table 7).

## 4. Discussion

The main objective of this quasi-experimental study was to measure its effectiveness in improving the management of nonspecific lower back pain in nonprofessional caregivers. Additionally, it was interesting to determine whether the intervention could improve other elements present in this type of caregiver. Furthermore, data were collected to investigate the profiles of this group compared to their sociodemographic characteristics.

The profile of caregivers identified in the sample, consisting of middle-aged women with primary education, closely resembles the profiles found in similar studies [38,39,40]. This phenomenon suggests consistency in sociodemographic patterns within this group and highlights the need to explore the variables that could influence these common characteristics more deeply.

It is crucial to analyse how factors such as age, marital status, education level, and source of income interact with each other and how these aspects can influence various situations in the lives of caregivers, from their family decisions to their socioeconomic outlook. It is noteworthy that almost half of the participants considered taking on the role of caregiver as their only option. Although nonprofessional caregivers are generally involuntarily thrust into this responsibility [41], they are often the only available family member or the closest relative to the dependent person. This phenomenon is prevalent in Spanish society, where the family care of older and/or dependent individuals is still deeply rooted, more because of tradition or culture than for economic reasons [42].

In addition, the nonprofessional caregiving time shown by the participants, exceeding two years in 80% of the cases, demonstrated a high level of involvement and dedication from the sample. This, combined with the high demand for complex care for a group of individuals with severe or moderate dependency in over 60% of cases, could explain not only the presence of lumbar pain in these caregivers but also the high rates of caregiver burden. According to Fujihara et al. [43], high caregiver burden indices are expected for long-term family caregivers with significant dependents under their care, as both aspects are considered predictors of caregiver burden. This only serves to immerse the caregiver in a situation where the burden of care impacts their well-being and functional state, physically, psychologically, socially, and spiritually [44].

All participants experienced lower back pain and showed a moderate baseline level of pain that caused moderate disability in many cases, which is consistent with other studies [14,40]. This finding may suggest that the group had a manageable level of impairment. However, if baseline results of caregiver burden (where the physical component has a specific weight) and health-related quality of life (where caregivers showed more problems in the dimensions of pain and mobility) were observed, the direct influence of lower back pain on the daily lives of nonprofessional caregivers can be inferred. In this regard, the results align with those of Vega-Vélez et al. [45] and may indicate an association between factors inherent to nonprofessional caregiving and the onset of musculoskeletal pain, such as lower back pain.

Pain is one of the most disabling and common causes of disability among nonprofessional caregivers. The results highlight its significant impact on the quality of life of this group, generating a confluence of additional problems, among which anxiety stands out. The interaction between chronic pain and anxiety forms a complex scenario that could explain why caregivers perceive a progressive deterioration in their health, manifested as notably diminished scores. This association between pain, anxiety, and negative perception of health aligns with the findings of previous studies, as noted by Rodríguez-González et al. [46]. The cumulative burden of these two variables, physical and emotional, can have a substantial impact on the overall well-being of nonprofessional caregivers. The daily experience of dealing with pain, which is often chronic, along with the anxiety associated with caregiving responsibilities contributes to a cycle that perpetuates the perception of an increasingly weakened state of health.

### 4.1. Effectiveness of the TRANSFE Program for Lower Back Pain Management

Lower back pain was studied in three dimensions, encompassing presence, level, and associated disability, which were the main focus of the TRANSFE program, with the purpose of thoroughly analysing its impact on the management of this condition. The consideration of this variable as the key to assessing the specific effectiveness of the program was based on the need to effectively address the challenges faced by nonprofessional caregivers in relation to lower back pain. It is important to note that although this was one of the inclusion criteria for this study, all nonprofessional caregivers presented with non-specific lower back pain. A notable aspect in line with the hypothesis raised is that lower back pain is a condition commonly observed in this group [14,22,23].

The significantly higher frequency of nonspecific lower back pain compared to the general population average in Spain [47] suggests the need for personalised intervention strategies adapted to the unique circumstances of nonprofessional caregivers. This differentiated approach may be crucial to improving the quality of life of these caregivers and, at the same time, contribute to the overall effectiveness of the TRANSFE program in managing lower back pain in this specific group.

The results obtained reveal that the TRANSFE program succeeds in consistently improving lower back pain in the participants across all three dimensions considered. Although the literature evaluating educational interventions in patient mobilisation and transfer techniques is limited, previous studies have supported the effectiveness of such interventions by demonstrating significant improvements in caregivers [27,28,48].

The positive impact of the TRANSFE program on all evaluated dimensions suggests that it is effective in terms of the assessed time period. However, it is essential to note that the duration of these effects beyond 3 months could not be confirmed with certainty in this study. While some studies suggest that the improvement in lower back pain can be long-lasting, even extending beyond 12 months [14,49], additional research is needed to validate the persistence of the long-term benefits of the TRANSFE program.

The evaluation of the program’s effectiveness was based on the results obtained by the caregivers on validated scales at 3 months; however, we did not observe whether the caregivers performed these techniques as taught in the program, so it cannot be confirmed that they were performed correctly. Nonetheless, the result of the statistical analyses was so compelling that it is presumable that the caregivers correctly acquired the skill in these techniques, and as indicated by other authors [26,28,29], despite the lack of direct observation of the application of ergonomic techniques, the results suggest greater confidence and efficiency in their tasks, leading to significant improvement in lower back pain. However, to obtain a more precise understanding of the program’s effectiveness, it would be beneficial to implement future evaluations that include direct observations. This would confirm that the program improves the caregivers’ abilities not only in the short term but also in a lasting manner.

### 4.2. Effectiveness of the TRANSFE Program Compared with Other Variables

The high prevalence of baseline levels of caregiver burden highlights the magnitude of this problem, especially among nonprofessional caregivers, as supported by the literature [15,16,17,18,19,20,21]. The emotional burden associated with nonprofessional caregiving adds an additional dimension to this challenge, contributing significantly to the burden experienced by these caregivers.

The analyses indicated that the TRANSFE program was as effective as the other interventions [24,25,50] at reducing caregiver burden. This finding suggests that these interventions play a crucial role in the program’s effectiveness regardless of whether the content focuses exclusively on emotional issues [51,52] or addresses, as is the case with TRANSFE, a modification of lower back pain [29,33]. In this regard, the program’s ability to positively influence caregiver burden emphasises the importance of adopting a comprehensive approach to these interventions, addressing both physical and emotional aspects.

It is generally observed in the literature that interventions aimed at nonprofessional caregivers tend to achieve positive results in terms of perceived social support [53]. This study supports this trend by confirming the effectiveness of the TRANSFE program in improving perceived social support among participants and highlights the consistency of these results with previous evidence. This finding contrasts with the assertion of Palmar-Santos [54], who suggested that group interventions might perform better because of the sense of support generated by finding similar social situations. However, other studies [55] have indicated that improvements in emotional and social aspects can be achieved through individual and home-based interventions.

The discrepancy in the results highlights the complexity of social and emotional dynamics in the context of nonprofessional caregiving. The effectiveness of interventions may depend on various factors such as individual preferences, the specific nature of the challenges faced by each caregiver, and the surrounding social structure. This finding emphasises the importance of considering the diversity of approaches in the implementation of interventions to ensure that they are optimally adapted to the unique needs and circumstances of nonprofessional caregivers. However, as the study was not designed with a control group and the intervention was conducted in a group setting, it is challenging to determine whether the individualisation of content was crucial to the success of the results, both in terms of social support and other variables.

The results indicate that health-related quality of life experiences significant improvements, especially in dimensions inherently affected by nonprofessional caregivers, such as pain and anxiety, corroborating previous findings [15,20,21]. Additionally, there was a discrepancy in the self-perception of health status, suggesting that the intervention improved caregivers’ impressions of their health. Although there is a consensus in the literature that interventions tend to yield good results in the health-related quality of life of caregivers [50,51], this improvement is closely linked to other factors such as caregiver burden and social support. The complexity of these interrelationships highlights the importance of comprehensively addressing both the physical and emotional aspects of nonprofessional caregiving.

It is noteworthy that, although the TRANSFE program proved to be effective in managing lower back pain in caregivers, there is less consensus regarding these complementary variables. Improvements produced by interventions in nonprofessional caregivers are often intrinsically linked to the caregiver role and only change if the underlying cause that generates them disappears, as noted by Beinart et al. [56]. This underscores the need for personalised and sustainable approaches that address the inherent complexities of nonprofessional caregiving and its effects on caregivers’ quality of life.

Finally, it is important to note that during the implementation of the program sessions, no adverse situations occurred that affected its performance, caregivers, or dependent individuals. As documented in other studies on non-professional caregivers [25,28,40,57], no negative or hazardous effects were recorded, thus the program intervention can be considered safe for both caregivers and dependent individuals.

### 4.3. Limitations

This study was registered retrospectively, which can be considered a limitation of the work. At the beginning of the study, the authors were unaware of the International Committee of Medical Journal Editors (ICMJE) policy, which recommends the prospective registration of all intervention studies. Additionally, due to methodological issues, this work could not be designed as a randomized clinical trial since a control group could not be created, and the sample recruitment was carried out by nurses not affiliated with the research team, preventing exhaustive control over this process. Therefore, the results should be interpreted with caution, and no definitive conclusions can be drawn about the highlighted aspects, such as the individualization of the intervention, which may have been crucial for the program’s success by allowing content specificity. As soon as we became aware of this policy, the study was registered on the Open Science Framework platform (registration number 10.17605/OSF.IO/K7WTE).

One of the difficulties encountered in the development of this research was the recruitment of caregivers, as many potential participants suffered from lower back pain that could be associated with non-nonspecific causes and therefore could be related to reasons other than nonprofessional caregiving (which was an exclusion criterion). Although some of the studies used for the design of the intervention had more limited samples, it is true that their small size limits the generalisation of results beyond the individuals who participated in this study; therefore, this aspect suggests caution when interpreting the applicability of the findings and generates recommendations to expand the sample in future research.

Additionally, repeated measurements at 3 months were considered another limitation. This relatively short period may not be sufficient to comprehensively and accurately assess the temporal duration of the benefits of the proposed intervention. Some effects may require long-term follow-up to fully understand their persistence and to assess the sustainability of improvements observed in variables such as lower back pain, health-related quality of life, and health status perception. In the case of this research, it was decided not to extend the post-intervention measure beyond three months or conduct another measure later, as there was a group of dependent individuals of very advanced age and with such a delicate health status that the loss of participants throughout the study due to the death of dependent individuals was feared. Although the interventions on which the design of this intervention was based also had a measure at 3 months or less, we believe that the results would have been stronger if it had been possible to determine whether the positive effects were sustained beyond 3 months.

### 4.4. Future Directions of Research and Application to Clinical Practice

Considering the limitations identified in this study, it would be valuable to conduct additional research to address them. A more robust study design with a larger sample size, long-term follow-up, and a control group would allow for more generalisable results and provide a more comprehensive understanding of the effectiveness of the TRANSFE program in managing lower back pain and other aspects related to nonprofessional caregivers.

Additionally, incorporating a cost-effectiveness analysis is crucial for assessing the economic viability of implementing the TRANSFE program in the healthcare system. This approach would not only demonstrate clinical effectiveness but also calculate benefits in relation to the costs associated with the intervention, providing valuable information for healthcare decision-makers to determine the cost-effectiveness and practical utility of the program in the healthcare context.

On the other hand, it would be interesting to implement educational programs on ergonomics in mobilization and transfer for all people who care for dependent individuals before the onset of lower back pain. Including this educational component could significantly contribute to the prevention of injuries and an overall improvement in the quality of care provided. In future versions of the TRANSFE program, we plan to integrate these ergonomic education elements to ensure that all caregivers have the necessary tools to perform their tasks safely and efficiently.

## 5. Conclusions

The results provide evidence of the immediate effectiveness of the TRANSFE program in improving lower back pain in nonprofessional caregivers. Additionally, the intervention demonstrated statistically significant improvements in health perception and quality of life, which is crucial considering the emotional burden associated with nonprofessional caregiving, as well as additional improvements in aspects generally present in these caregivers, such as high caregiver burden and low perceived social support. However, given the inherent limitations of the study, and the type of design, it is necessary to interpret these results with caution when asserting their scope. The small sample size recruited and the follow-up period underscore the need for additional research to consolidate and generalize the results. It is suggested to expand the sample size and extend the long-term follow-up period to assess the persistence of the benefits obtained. Incorporating a cost-effectiveness analysis could further strengthen the foundation for the implementation of the TRANSFE program in the healthcare system.

## Figures and Tables

**Table 1:** Characteristics and psychometric properties of measurement instruments.

	**Presence of lower back pain**
Instrument	Back Pain Index [30]
Characteristics	Assess the physical abilities of the patient with lower back pain. Perform 5 movements, with a score ranging from 0 to 3. Total range: 0–15.
Psychometric properties	Test–retest reliability: 0.97. Construct validity: good correlation between the Back Pain Index, Oswestry, and McGill Pain Questionnaire. Sensitivity: 95%.
	**Level of lower back pain**
Instrument	Visual Analogue Scale [31]
Characteristics	Rule drawn with a range between 0 (no existence of pain) and 10 (the worst imaginable pain).
Psychometric properties	Test–retest reliability: 0.95. Internal consistency reliability: 0.97.
	**Degree of disability due to lower back pain**
Instrument	Oswestry Disability Index [32]
Characteristics	Measures the limitations caused by lower back pain on daily activities. Ten questions, 6 possible responses. 5 levels of stratification.
Psychometric properties	Test–retest reliability: 0.99. Internal consistency reliability: 0.85. Construct validity: good correlation with SF-36 and multidimensional pain inventory.
	**Level of caregiver burden**
Instrument	Caregiver Burden Scale/Zarit Questionnaire [34]
Characteristics	Measures the degree of emotional and physical exhaustion of caregivers. Twenty-two questions, scored from 1 to 5. Range: 22–110. Three levels of stratification.
Psychometric properties	Test–retest reliability: 0.86. Internal consistency reliability: 0.91. Construct validity: good correlation between Zarit, General Health Questionnaire, and Katz. Sensitivity: 93.3%.
	**Perceived social support**
Instrument	Duke-UNK Questionnaire [35]
Characteristics	Multidimensional measurement of social support: confidential support and emotional support. Eleven questions scored from 1 to 5. Two levels of stratification.
Psychometric properties	Test–retest reliability: 0.90. Internal consistency reliability: 0.92. Construct validity: several predictions were tested. Significant differences were found based on education level, age, and type of caregiving.
	**Health-related quality of life**
Instrument	EuroQol-5D Scale [36]
Characteristics	Generic instrument with 5 dimensions, each with 3 possible responses, current health status with 3 possible responses, and a visual analogue scale from 0 to 100 for health level.
Psychometric properties	Reliability of internal consistency: 0.64. Validity: tested across various pathologies, demonstrating the instrument’s validity in different groups. Sensitivity: has shown sensitivity to changes in health status in various patient groups.

**Table 2:** Sociodemographic profile of nonprofessional caregivers and dependent individuals.

**Sociodemographic profile of dependent individuals**		
Sex and Age	n (%)	X ± DE
Female	24 (61.5%)	79.75 ± 9.92
Male	15 (38.5%)	72.46 ± 12.88
Total	39 (100.0%)	76.94 ± 11.54
Level of dependence (Barthel)	Frequency (n)	Percentage (%)
Severe (20–35)	17	43.60
Moderate (40–50)	9	23.10
Limited (>60)	8	20.50
Complete (<95)	4	10.30
Independent (100)	1	2.60
Total	39	100.00
**Sociodemographic profile of nonprofessional caregivers**		
Sex and Age	n (%)	X ± DE
Female	26 (72.2%)	60.03 ± 10.30
Male	10 (27.8%)	58.00 ± 10.40
Total	36 (100.00%)	59.75 ± 10.20
Educational level	Frequency (n)	Percentage (%)
Primary Education	16	44.44
Secondary Education	15	41.70
Higher Education	5	19.00
Illiterate	0	0.00
No formal studies	0	0.00
Total	36	100.00
Time as a caregiver		
>24 months	30	83.30
12–24 months	2	5.60
6–12 months	4	11.10
Total	36	100.00
Reason for being a nonprofessional caregiver		
Only option	17	47.20
Voluntary decision	13	35.10
The family decided	5	13.90
The dependent person decided	1	2.80
Total	36	100.00

**Table 3:** Baseline data on the following variables: presence of lumbar pain, level of lumbar pain, caregiver burden, perceived social support, and health-related quality of life.

	Average	Standard Deviation	95% Confidence Interval of the Difference		N
			Lower	Upper	
IDE PRE-IE	7.12	3.67	0	13	36
EVA PRE-IE	5.69	2.65	0	9	36
ZARIT PRE-IE	71.91	21.28	47	100	36
UNK PRE-IE	32.36	7.88	15	45	36
EQ EVA PRE-IE	6.25	1.48	2	9	36

IDE PRE-IE: Presence of lower back pain pre-intervention; EVA PRE-IE: Lower back pain level pre-intervention; ZARIT PRE-IE: Caregiver burden pre-intervention; UNK PRE-IE: Perceived social support pre-intervention; EQ EVA PRE-IE: Visual analogue scale for the current health state in EuroQol-5D.

**Table 4:** Paired differences in the presence and level of lumbar pain in all caregivers before and after the intervention.

	Average	Standard Deviation	95% Confidence Interval of the Difference		Wilcoxon Test	N	*p*-Value
			Lower	Upper			
EVA/1–EVA/2	1.8182	2.0533	1.0901	2.5462	5.087	33	<0.001
IDE/1–IDE/2	2.8788	3.2573	1.7238	4.0338	5.077	33	<0.001

EVA/1, lumbar pain level before intervention; EVA/2: Lumbar pain level after intervention; IDE/1: Presence of lumbar pain before intervention; IDE/2: Presence of lumbar pain after intervention.

**Table 5:** Disability due to lumbar pain before and 3 months after the intervention.

Kappa = 0.275		OSWESTRY POST-IE				
		NR	INTENSE	MINIMAL	MODERATE	Total
OSWESTRY PRE-IE	INTENSE	0	2	2	7	11
	MINIMAL	1	0	9	0	10
	MODERATE	2	0	6	7	15
	Total	3	2	17	14	36

OSWESTRY PRE-IE: Disability due to lumbar pain before intervention; OSWESTRY POST-IE: Disability due to lumbar pain after intervention; NR: No response.

**Table 6:** Caregiver burden and perceived social support before and after 3 months of intervention in the caregiver sample.

**Caregiver Burden**					
Measurement	N	Average	Standard deviation	Wilcoxon Test	*p*-value
ZARIT PRE-IE	36	66.27	21.24	−2.322	0.020
ZARIT POST-IE	33	55.54	10.11		
**Perceived Social Support**					
Measurement	N	Average	Standard deviation	Wilcoxon Test	*p*-value
UNK PRE-IE	36	32.24	7.80	−5.75	<0.001
UNK POST-IE	33	35.72	6.29		

ZARIT PRE-IE: pre-intervention measurement of caregiver burden; ZARIT POST-IE: post-intervention measurement of caregiver burden; UNK PRE-IE: pre-intervention measurement of perceived social support; UNK POST-IE: post-intervention measurement of perceived social support.

**Table 7:** Results of the health-related quality of life variable before and after 3 months of the intervention.

Dimensions					
	Care	Activities	Pain	Anxiety	Mobility
	K = 0.412	K = 0.212	K = 0.153	K = 0.084	K = 0.365
**Perceived Health Compared to 12 Months Ago**					
K = 0.429					
**Self-Perception of Current Health Status**					
Measurement	N	Average	Standard deviation	Wilcoxon Test	*p*-value
EQ EVA PRE-IE	36	6.250	1.480	−1.822	0.068
EQ EVA POST-IE	33	6.788	1.082		

EQ VAS PRE-IE: pre-intervention measurement of self-perception of current health status; EQ VAS POST-IE: post-intervention measurement of self-perception of current health status.

## Data Availability

The data presented in this study are available on request from the corresponding author due to privacy reasons.

## Public Involvement Statement

No public involvement in any aspect of this research.
